# Engineering Neurotoxin-Functionalized Exosomes for Targeted Delivery to the Peripheral Nervous System

**DOI:** 10.3390/pharmaceutics16010102

**Published:** 2024-01-12

**Authors:** Mena Asha Krishnan, Olawale A. Alimi, Tianshu Pan, Mitchell Kuss, Zeljka Korade, Guoku Hu, Bo Liu, Bin Duan

**Affiliations:** 1 Mary and Dick Holland Regenerative Medicine Program, University of Nebraska Medical Center, Omaha, NE 68198, USA; mekrishnan@unmc.edu (M.A.K.); oalimi@unmc.edu (O.A.A.); tpan@unmc.edu (T.P.); mitchell.kuss@unmc.edu (M.K.); 2 Division of Cardiovascular Medicine, Department of Internal Medicine, University of Nebraska Medical Center, Omaha, NE 68198, USA; 3 Department of Pediatrics, University of Nebraska Medical Center, Omaha, NE 68198, USA; zeljka.korade@unmc.edu; 4 Child Health Research Institute, Omaha, NE 68198, USA; 5 Department of Biochemistry and Molecular Biology, University of Nebraska Medical Center, Omaha, NE 68198, USA; 6 Department of Pharmacology and Experimental Neuroscience, University of Nebraska Medical Center, Omaha, NE 68198, USA; guoku.hu@unmc.edu; 7 Department of Surgery, University of Nebraska Medical Center, Omaha, NE 68198, USA; 8 Department of Mechanical and Materials Engineering, University of Nebraska Lincoln, Lincoln, NE 68588, USA

**Keywords:** red blood cells, tetanus toxin-C fragment, click chemistry, target delivery, peripheral nerve injury

## Abstract

The administration of therapeutics to peripheral nerve tissue is challenging due to the complexities of peripheral neuroanatomy and the limitations imposed by the blood–nerve barrier (BNB). Therefore, there is a pressing need to enhance delivery effectiveness and implement targeted delivery methods. Recently, erythrocyte-derived exosomes (Exos) have gained widespread attention as biocompatible vehicles for therapeutics in clinical applications. However, engineering targeted Exos for the peripheral nervous system (PNS) is still challenging. This study aims to develop a targeted Exo delivery system specifically designed for presynaptic terminals of peripheral nerve tissue. The clostridium neurotoxin, tetanus toxin-C fragment (TTC), was tethered to the surface of red blood cell (RBC)-derived Exos via a facile and efficient bio-orthogonal click chemistry method without a catalyst. Additionally, Cyanine5 (Cy5), a reactive fluorescent tag, was also conjugated to track Exo movement in both in vitro and in vivo models. Subsequently, Neuro-2a, a mouse neuronal cell line, was treated with dye-labeled Exos with/without TTC in vitro, and the results indicated that TTC-Exos exhibited more efficient accumulation along the soma and axonal circumference, compared to their unmodified counterparts. Further investigation, using a mouse model, revealed that within 72 h of intramuscular administration, engineered TTC-Exos were successfully transported into the neuromuscular junction and sciatic nerve tissues. These results indicated that TTC played a crucial role in the Exo delivery system, improving the affinity to peripheral nerves. These promising results underscore the potential of using targeted Exo carriers to deliver therapeutics for treating peripheral neuropathies.

## 1. Introduction

Peripheral neuropathies are neurological disorders that commonly afflict the peripheral nerves. With a prevalence rate of 1% in adults and increasing to 7% after 55 years of age, they represent a significant public health concern worldwide [1,2]. The causes of peripheral neuropathies are diverse and encompass a wide range of factors, including traumatic injuries or nerve compression [3,4], diabetes mellitus, which affects between 25% to 50% of diabetics [5], exposure to toxins such as drug- and chemotherapy-induced neuropathy [6], infectious diseases [7,8], and vitamin imbalances [9]. The condition’s pathophysiology can include damage to small or large-diameter nerve fibers, neuronal cell bodies, and axons, as well as demyelination or an association of these factors. This clinically manifests in neuropathic pain, hypoesthesia, motor deficits, ataxia, areflexia, and paralysis in severe cases [10,11].

Commonly administered medications to alleviate pain and manage associated symptoms include calcium channel blocker gabapentenoids, tricyclic antidepressants, and selective serotonin–norepinephrine reuptake inhibitors (SNRI) as first-line options [12,13]. Unfortunately, no treatment addresses the repairing of damaged nerves, creating a need for strategies that focus on axonal regeneration, re-myelination, and anti-inflammation. Several neurotrophic factors (brain-derived neurotrophic growth factor (BDNF) [14], nerve growth factor (NGF) [15,16], and neurotrophin-3 [17]) have shown promise in pre-clinical investigations by promoting synaptic plasticity along with neurite and axonal growth [18]. Extensive research on delivering nucleic acid-based pro-drugs via non-viral vectors to modulate the local expression of neurotrophins and promote functional recovery has also been conducted [19,20]. However, low systemic half-life, pleiotropic effects, reduced cargo size, immunogenic incompatibility, and toxic side effects of large doses hamper further clinical success [21,22].

Moreover, the delivery of these therapeutics to peripheral nerve tissue remains a complex undertaking due to the intricacies of peripheral neuroanatomy. The constraints posed by the blood–nerve barrier (BNB) also make drug targets inaccessible [23]. The sciatic nerve is particularly impervious to hydrophilic molecules and larger polypeptides, permitting only small lipophilic molecules and limiting therapeutic strategies [24,25]. Furthermore, the perineural and endoneural layers restrict the delivery of local analgesics that relieve neuropathic pain [26].

Targeted drug delivery is a promising approach to address challenges associated with conventional treatments. It involves functionalizing the drug carrier with a ligand that specifically binds to a receptor expressed in nervous tissue. This enhances the concentration and distribution of the drug in the target area, while also extending its circulation time [27].These factors increase the efficacy of the administered dose, reduce common side-effects, and mitigate toxicity [28]. Current methods that employ Tet1, a 12-amino acid peptide, to transport nucleic acids complexed with various polymers, such as polyethyleneimine [29], N-(2-hydroxypropyl) methacrylamide (HPMA) [30], and poly(E-caprolactone)-block-poly(ethylene glycol) (PEG-b-PCL) [31], have restrictions in renal clearance and loading capacity. Modified dioleoyl-phosphatidylcholine (DOPC) liposomes with non-ionic emulsifier Poloxamer 188 and cholesterol-mimicking nerve cell membrane have also shown beneficial results, however, safer and more efficient cargo vehicles are required [32].

Recently, erythrocyte-derived exosomes (Exos) have emerged as safe and cell-friendly vehicles for delivering therapeutics [33,34]. These membranous nanovesicles, originating from the endosomal compartment inside cells, mediate intercellular communication and offer several advantages, including (i) innate safety and stability [35], (ii) low immunogenicity [36], and (iii) the ability to easily modify chemical moieties on their surface [37]. These characteristics make them ideal for delivering therapeutics to nerve tissues via target-specific cues, thereby facilitating regeneration and repair. One significant advantage of using red blood cells (RBCs) to derive Exos is their abundance and high availability in blood banks, as well as their ability to be acquired from patient transfusions for personalized therapy [36]. Moreover, RBCs generate a high yield of Exos (10^12^ Exos/mL from 100 million RBCs), and easy culture protocols allow for scalability. Additionally, enucleated RBCs are devoid of DNA, thus minimizing the risks of genetic transfer [38]. However, to achieve efficient drug delivery, it is vital to modify the surfaces of Exos to target specific tissues. Such modifications enable Exos to interact rapidly with affected tissues and localize there, reducing systemic clearance [39].

Herein we propose a simple and innovative Exo-based targeted delivery platform that leverages the binding of bacterial neurotoxins to presynaptic terminals of peripheral nerve tissue to confer target specificity [40,41]. Our approach involves attaching a tetanus toxin-C fragment (TTC), which is a nontoxic fragment of the tetanus toxin from the clostridium neurotoxin family, onto the RBC-derived Exo surface via a simple and efficient bio-orthogonal click chemistry. This novel platform aims to address three issues: (i) protect drug cargo and increase circulation time, (ii) recognize and accumulate in target nerves, (iii) cross the BNB. The effectiveness and uptake efficiency of the engineered TTC-modified Exos (TTC-Exo) were assessed with in vitro experiments using Neuro-2a cell lines. The Exos were then administered into a mouse model via intra-muscular injection, allowing for natural retrograde transport mechanisms to cross the BNB and reach peripheral nerves. After 72 h, it was discovered that the Exos had accumulated in the sciatic nerve. These promising findings demonstrate the potential of utilizing targeted Exo carriers to deliver therapeutics for treating peripheral neuropathies.

## 2. Materials and Methods

### 2.1. Materials

The details of the materials used in the experiments are detailed as follows: Calcium Ionophore (A23187; Sigma-Aldrich; St. Louis, MI, USA), Dulbecco’s Phosphate Buffer Saline (DPBS) modified without Ca^2+^ and Mg^2+^ (SH30028.03; Hyclone; Cytiva; Logan, UT, USA), Eagle’s Minimum Essential Medium (EMEM) growth medium (112-016-101; Quality Biologicals; Gaithersburg, MD, USA), Penicillin Streptomycin (5000 U/mL; 150-700-63; Gibco; Waltham, MA, USA), Fetal bovine serum (FBS) (160-000-44; Gibco; USA), trypsin-Ethylenediamine tetraacetic acid (EDTA) (25200-072; Gibco; USA), GlutaMAX Sup-plement (35050061; ThermoFisher Scientific; Waltham, MA, USA), Tetanus toxin C-fragment from *Clostridium tetanii* (23222; Cayman Chemical; Ann Arbor, MI, USA), Sulfo-Cyanine5 Azide (Sulfo-Cy5-N_3_) (22483; Broadpharm; San Diego, CA, USA), Dibenzocyclooctyne-(Polyethylene glycol)4-N-hydroxysuccinimidyl ester (DBCO-PEG4-NHS) ester (22288; Broadpharm; USA), Azido-(Polythylene glycol)_5000_-N-hydroxysuccinimidyl ester (Azido-PEG_5000_-NHS ester) (BP-21659; Broadpharm; USA), Poly-D-Lysine (A-38904-01; Gibco; USA), Valproic acid sodium salt (676380; Sigma Aldrich; USA); Paraformaldehyde (P6148; Sigma-Aldrich; USA), Optimal cutting temperature (OCT) Compound (4585; Fisher HealthCare; Waltham, MA, USA), RIPA (Radioimmunoprecipitation Assay) lysis buffer (89900; ThermoFisher Scientific; USA), PKH26 lipophilic labeling dye (PKH26GL; Sigma-Aldrich; USA). The details of the antibodies used are as follows: Anti-Neurofilament 200 (N4142; Sigma-Aldrich; USA), anti-Tubulin β-3 (TUJ1) (MMS-435P; Biolegend; San Diego, CA, USA), Anti-Synaptophysin (SY38) (ab8049; Abcam; Waltham, MA, USA); Anti-CD9 (ab223052; Abcam; USA); Anti-ALIX (3A9) (ab117600; Abcam; USA); Anti-Calnexin (C7617; Sigma-Aldrich; USA)

### 2.2. Cell Culture

The Neuro-2a cell line (CC131; ATCC) was cultured in EMEM growth medium supplemented with 1% penicillin–streptomycin, 10% fetal bovine serum, and 2 mM glutamine until 80% confluent. For inducing differentiation, the cells were seeded at a density of 20,000 cells/well in a 24-well plate in complete growth medium overnight. The spent medium was then replaced with EMEM supplemented with 1% penicillin-streptomycin, 1% FBS, 2 mM glutamine, and 1 mM valproic acid. The cells were maintained in these conditions for 72 h to allow for differentiation.

### 2.3. Exo Isolation and Purification

Human RBCs (1 × 10^8^ cells/5 mL) were obtained from a single healthy donor patient for each batch from the elutriation core and washed with ice-cold 1× DPBS through centrifugation at 2000× *g* and 4 °C for 10 min. RBCs were collected in batches from a total of 6 healthy donor patients throughout the entire study. The cells were then washed with ice-cold 1× DPBS and treated with 20 mM calcium ionophore in 1× DPBS at 4 °C for 12 h. The protocol for isolation and purification is adapted from a published method [42]. Briefly, Exos were isolated by removing the cellular debris and pellet through centrifugations at 2000× *g* and 4 °C for 10 and 20 min then 12,000× *g* for 30 min. The supernatant was filtered, and the Exos were concentrated through ultracentrifugation at 110,000× *g* and 4 °C for 70 min. The Exo pellet was purified through repeated ultracentrifugation, and the final product was resuspended in 1× DPBS and stored at 4 °C for up to a week or at −80 °C for up to 1–2 months.

### 2.4. Characterization of Exos

The sizes, concentration, and distribution of the purified Exos were quantified by nanoparticle tracking analysis (NTA), using the NanoSight (NS300) instrument (Malvern Panalytical Inc., Westborough, MA, USA).

The Exo surface morphology was determined by transmission electron microscopy (TEM, Hitachi H7500; Hitachi America, Ltd., Santa Clara, CA, USA) as described previously [42]. Additionally, the surface charge was also measured using a Zetasizer Nano (Malvern Panalytical Inc., Westborough, MA, USA).

#### Western Blot

Purified Exo samples were initially lysed in RIPA lysis buffer with protease inhibitor (100:1). Protein concentrations were assayed using a Micro BCA protein assay kit (23235; Thermo Scientific) and normalized prior to separation. A total of 40 µg of protein was loaded and separated on a 10% SDS-polyacrylamide protein gel and Precision Plus Protein Dual Color Standards^TM^ ladder (1610374; BioRad Laboratories Inc., Hercules, CA, USA) and was used as a reference for molecular weight determination. The separated proteins were transferred onto a nitrocellulose blotting membrane, which was divided into four pieces. The membrane was then blocked with 5% skimmed milk powder in Tris-buffered saline with 0.1% Tween-20 (TBST) for 30–60 min at room temperature with gentle shaking. The blot was then incubated overnight at 4 °C with primary antibodies against CD9 (1:1000, Abcam), Alix (1:1000, Abcam), and calnexin (1:1000, Sigma) in 1% TBST. After three washes with TBST, the blot was incubated with HRP-conjugated anti-rabbit and anti-mouse 2° (Abcam 1:10,000) for 2 h at room temperature with gentle shaking. The blot was finally developed using the Gel Doc™ (Bio-Rad, USA) imaging system.

### 2.5. Conjugation of TTC-Fragment and Exo Surface Labeling

The TTC fragment was first modified with PEG_5000_-N_3_. The TTC (10 µg; 0.2 mmol) was reacted with N_3_-PEG_5000_-NHS (21 µg; 0.53 µmol) overnight at 4 °C, and then the TTC-PEG_5000_-N_3_ was washed and purified using ultra-filtration columns.

Purified and isolated Exos suspended in 100 µL 1× DPBS (1.0–1.8 × 10^12^ particles/mL) were reacted with 100 µL of 36 µM of DBCO-NHS-ester for 3 h at room temperature (RT). Afterwards, unreacted DBCO-NHS-ester molecules were removed by washing them through centrifugation at 7500× *g* via Nanosep^®^ 100 K (Pall Life Sciences, Port Washington, NY, USA) ultrafiltration columns, three times [43].

For TTC-Exo preparation, the DBCO-conjugated Exos were reacted with TTC-PEG_5000_-N_3_ (2 µL; 0.36 µM) at RT for 3 h. Finally, Sulfo-Cy5-N_3_ (3 µL; 36 µM) was added to the reaction and stirred at RT for 1 h. The ratio of DBCO-NHS-ester/Cy5-N_3_/TTC-PEG_5000_-N_3_ molecules was maintained at 400:400:1. The remaining unreacted molecules were left to react at 4 °C for 16 h. The samples were again washed in 1× DPBS and concentrated using centrifugation at 7500× *g* via Nanosep^®^ 100 K (Pall Life Sciences) ultrafiltration columns, three times. The purified product was stored at 4 °C for up to a week.

### 2.6. Staining of Exos with Lipophilic Dye

Purified DBCO-conjugated Exos (1 × 10^8^ particles/mL) were stained with 2 µM PKH26 lipophilic dye and incubated in the dark at room temperature for 10 min. Excess dye was removed by washing them three times using 7500× *g* via Nanosep^®^ 100 K (Pall Life Sciences) ultrafiltration columns.

### 2.7. Uptake of TTC-Targeted and Unmodified Exos in Cells

Neuro-2a cells were seeded onto Poly-D-Lysine-coated glass coverslips in a 24-well plate at a density of 20,000 cells/well. After 72 h of differentiation, the neurite growth was observed. The cells were then treated with TTC-Exo and unmodified Exo (Un-Exo) samples (2.0 × 10^11^ particles/mL) and incubated overnight at 37 °C. The wells were washed twice with 1× DPBS and then fixed in 4% paraformaldehyde (PFA) at 4 °C for 4 h, followed by two additional washes with 1× DPBS. The cells were permeabilized with 0.2% Triton X-100 at room temperature for 10 min. After blocking with 1% bovine serum albumin (BSA) in 1× DPBS at 4 °C for 2 h, the cells were incubated overnight at 4 °C with a primary antibody against TUJ1 (1:400; Abcam) in 1% BSA with Triton X-100. After washing with 1× DPBS three times, the cells were incubated with a secondary antibody (1:500; Alexa488; Abcam) for 2 h at room temperature. The cells were washed again three times, and the cell nuclei were stained with DAPI (1:1000) for 15 min. The coverslips were gently mounted on imaging slides using Vectashield anti-fade mounting medium (Vector Laboratories Ltd., Newark, CA, USA), and images were captured using a Zeiss LSM 710 Confocal Laser Scanning Microscope (CLSM; Carl Zeiss Microscopy, LLC, White Plains, NY, USA).

### 2.8. Semi-Quantitative Imaging Analysis of CLSM Images

Semi-quantitative image analysis of the CLSM images of Neuro-2a cells incubated with Un-Exo and TTC-Exo samples was performed by using ImageJ version 1.54 software. The total fluorescence signal area in the red channel (Cy5 dye fluorescence) was measured. Three random images were analyzed per group. In the ImageJ version 1.54 software, the images were first scaled and converted to 8-bit versions. Under the “Set Measurements” option, the “area” and other parameters were set. Image thresholding was performed using the “Otsu” parameter to create a binary pixel image, and the values were measured and recorded. The final results were plotted in GraphPad Prism 9.5.0 software.

### 2.9. Animal Experiment Design and Treatment

The experiments involving animals were conducted in accordance with approved experimental protocols by the IACUC of UNMC. Six female mice (C57BL/6) aged 12 weeks were purchased from Charles River Laboratories, and the animals were labeled using ear tags and markings on their tails using permanent markers. The mice were randomly divided into two groups: Un-Exo and TTC-Exo, with three mice in each group, without any bias. The mice were weighed, anesthetized, and the hindleg and the flank regions were shaved prior to administration. Each mouse was injected with 1 × 10^12^ Exo particles/mL at the right hindlimb gastrocnemius muscle, with a total volume of 20 µL per animal.

### 2.10. Imaging and Bio-Distribution of Exos In Vivo

To examine the bio-distribution of Un-Exo and TTC-Exo, animals from both groups were monitored at four distinct time points following injection, namely 1-, 6-, 24-, and 72-h after using the in vivo Imaging System (IVIS^®^) Spectrum (Revvity Inc., Waltham, MA, USA) in vivo imaging system. At 24 h post-injection, one animal from each group was sacrificed, while the remaining ones were sacrificed at 72 h. Following their sacrifice, major tissues and organs, including the gastrocnemius muscle, bilateral sciatic nerve, spinal cord, heart, lung, liver, spleen, and kidneys, were collected for IVIS tissue imaging and histopathological analysis.

### 2.11. Immunofluorescent (IF) Staining and CLSM Imaging of Tissues

The tissues were fixed using a 4% PFA solution for 4 h at 4 °C, followed by sucrose solution overnight, before being embedded in OCT and cryosectioned with a thickness of 14 µm. For nerve sections, OCT was washed with PBS three times for 10 min, before the tissues were dehydrated in cold methanol for 10 min and washed again with PBS three times. The tissues were then permeabilized and blocked in a solution containing 5% goat serum in PBS with 0.2% Triton X-100 for 2 h at room temperature. Following blocking, the samples were incubated with a primary antibody against TUJ1 (1:400; Abcam) in the blocking solution overnight at 4 °C, followed by a secondary antibody (1:400; Alexa488) for 2 h, while avoiding light. After washing them with PBS, the samples were stained with DAPI (1:1000), mounted with antifade medium, and imaged using Zeiss LSM 710 CLSM.

To stain neuromuscular junctions, the gastrocnemius muscle was fixed with 4% PFA and split into longitudinal segments. The segments were then blocked and incubated with a primary antibody against neurofilament (1:200; Sigma-Aldrich) and synaptophysin (1:200; Abcam), followed by a secondary antibody (1:400; Alexa488) for 2 h. Finally, the tissues were imaged using a CLSM.

### 2.12. Statistical Analysis

All experiments were repeated at least thrice. All quantitative data are expressed as mean ± standard deviation (Mean ± SD). The data were first tested to fit a normal distribution using the Shapiro–Wilk and Kolmogorov–Smirnov tests. Statistical analysis was then performed using an unpaired t-test. A *p*-value less than 0.05 was considered significant for all statistical analyses. GraphPad Prism 9.5.0 software was used to perform statistical analysis.

## 3. Results

### 3.1. Isolation and Characterization of RBC-Derived Exos

**Figure 1:**
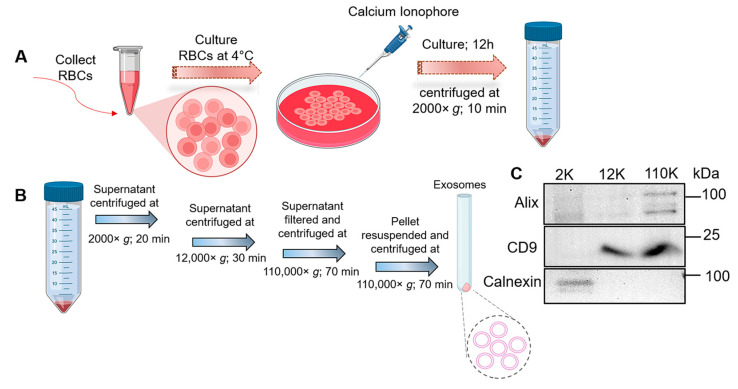
(**A**) Schematic showing the collection, culturing, and induction of RBCs with a chemical agent to produce Exos. (**B**) Schematic outlining the steps for isolation and purification of Exos by sequential centrifugation. (**C**) Western blot image showing protein markers positive (Alix and CD9) and negative (Calnexin) for Exos in samples collected after centrifugation at 2000× *g*, 12,000× *g*, and 110,000× *g* speeds. This schematic illustration was created using BioRender.com (accessed on 5 November 2023) with an authorized license.

The process used to produce and purify Exos from RBCs on a large scale is illustrated in Figure 1A. Incubation of RBCs (1 × 10^8^ cells) with calcium ionophore stimulates membrane budding and increases the release of extracellular vesicles [35,44]. The RBC-Exos were then isolated and purified through several centrifugation cycles. The first round removed the cell pellet and debris, while the latter two ultracentrifugation steps concentrated and purified the Exos (Figure 1B). The final yield after purification was found to be 1–2 × 10^12^ Exos/mL. The purified Exos were then analyzed for Exo-specific protein markers by western blot. Figure 1C shows the presence of the Exo-positive markers Alix and CD9 in the purified Exo lane (110,000× *g*). The endoplasmic reticulum marker calnexin was detected in the cell pellet (2000× *g* lane) but was absent in the Exo fraction, indicating that the Exo isolation procedure was well-optimized to remove contamination by cellular debris.

### 3.2. Design and Synthesis of TTC-Exo Targeted Delivery System for PNS

**Figure 2:**
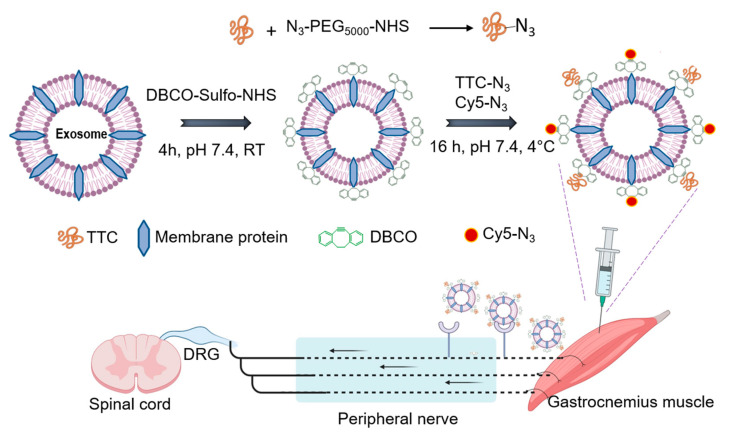
Schematic representation showing different steps for synthesizing TTC-targeted Exos along with a fluorescent Cy5 label through a click chemistry procedure. The schematic also shows the application of TTC-Exos as a targeted delivery system to the PNS after injection in the gastrocnemius muscle. This schematic illustration was created using BioRender.com (accessed on 15 November 2023) with an authorized license.

Subsequently, we prepared TTC-Exo to specifically target peripheral nerve tissues, as shown in Figure 2. The system involves a three-step reaction scheme. First, the bacterial tetanus toxin-C fragment was modified with a PEG_5000_-azide linkage. Then DBCO-Sulfo-NHS was used to crosslink the Exo surface with the DBCO moiety, forming stable covalent bonds with amine groups or phosphatidylethanolamine groups of Exo proteins [43]. Lastly, stable triazole linkages were formed by reacting DBCO functionalized Exos with the TTC-azide fragment via copper-free biorthogonal click chemistry. To visualize the Exos, Cy5-azide was conjugated onto the Exos. This click chemistry process allows for variable modification of Exo proteins with chemical moieties as per the clinical requirement and can be performed at a physiological pH in aqueous conditions without any catalysts. These conditions also make the system suitable for easy transition to in vitro and in vivo experiments. The TTC-Exo can be used to achieve targeted neuronal delivery to peripheral nerves via retrograde axonal transport mechanisms. Un-Exos were modified with Cy5-Azide moieties by the aforementioned procedure for visualization and comparison with the modified Exo group (TTC-Exo).

### 3.3. Characterization of TTC-Exo and Un-Exo

**Figure 3:**
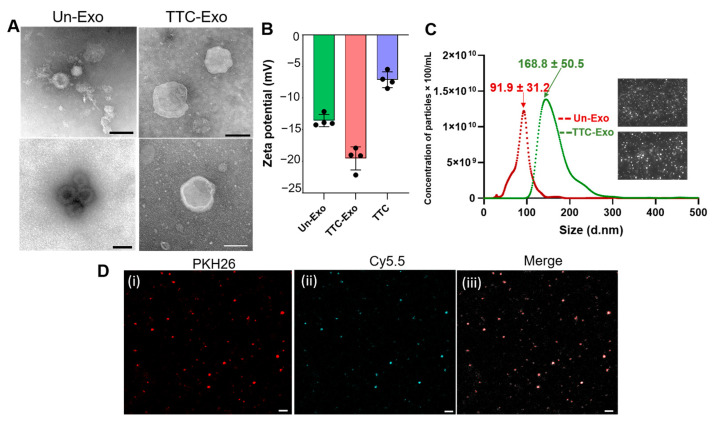
(**A**) TEM images showing the morphology of Un -Exo and TTC-Exo samples (Scale bar = 100 nm). (**B**) Zeta potential measurement of Un-Exo, TTC-Exo, and TTC protein samples (*n* = 4), data presented as mean ± SD. (**C**) NTA measurement of size (nm) and distribution (particle concentration × 100/mL) of Un-Exo and TTC-Exo (*n* = 3). Inset images show stills from video captures of Exo samples. (**D**) CLSM images of (i) unmodified, (ii) Cy5.5-azide conjugated protein, and (iii) co-localized images of Cy5.5 and PKH26 labelled Exo via click chemistry (Scale bar = 2 µm).

The morphology of the Exo samples, comprising both TTC-Exo and Un-Exo varieties, was initially examined using TEM, and the results are presented in Figure 3A. The TEM images indicated that both sets of Exos had cup-like, membranous structures. The TTC-Exo sample exhibited a general increase in diameter when compared to the Un-Exo sample. The surface potential was also measured, revealing an increase in negative potential from −13.5 ± 0.9 mV to −19.5 ± 1.8 mV (Figure 3B). Additionally, the particle concentration and size distributions were analyzed, confirming an increase in size from 91.9 ± 31.2 nm in the Un-Exo sample to 168.8 ± 50.5 nm in the TTC-Exo group, likely as a result of functionalization with TTC-PEG_5000_-N_3_ molecules (Figure 3C). Finally, the success of the click reaction in the conjugating groups on the Exo surface was confirmed through the visualization of PKH26 stained Exos functionalized with DBCO (Figure 3D(i)) and Cy5.5-azide conjugated protein fragment (Figure 3D(ii)) via CLSM, as shown in the merged image (Figure 3D(iii)).

### 3.4. Uptake of Un-Exo and TTC-Exo in Neuro-2a Cells

**Figure 4:**
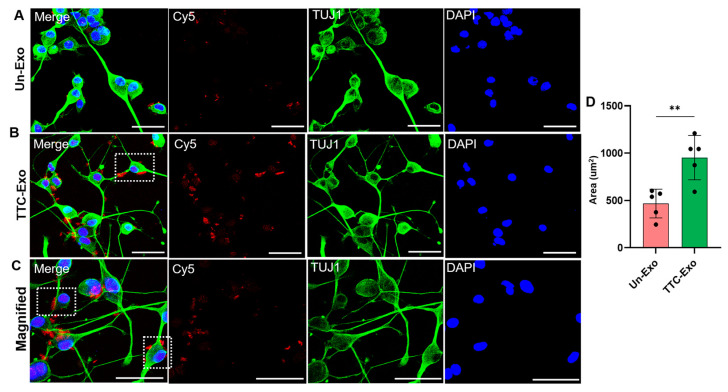
Cellular uptake and distribution of labeled Exos in Neuro-2a cell lines. (**A**) CLSM images showing IF staining for TUJ1 (green), Un-Exo (red), and nuclei staining (blue). (**B**) CLSM images showing IF staining for TUJ1 (green), TTC-Exo (red), and nuclei staining (blue). Dashed white boxes indicate accumulation of TTC-Exos on cell soma and axons. (**C**) Magnified (×2.5 zoom) CLSM images showing IF staining for TUJ1 (green), TTC-Exo (red), and nuclei staining (blue), with dashed white boxes emphasizing the distribution of labeled Exos along the neuronal axons. (**D**) Semi-quantitative analysis of the red signal (Cy5) area (µm^2^) measured in the CLSM images after incubation with Un-Exos and TTC-Exos, (*n* = 5). ** *p* < 0.01.

The aim of this study was to examine the distribution of Un-Exo and TTC-Exo in Neuro-2a cells using CLSM. Each well received a median dosage of 1 × 10^11^ Exos/mL and was incubated overnight. IF staining of TUJ1, which is implicated in neurogenesis, axon growth and maintenance, was performed to detect its expression in the PNS and central nervous system neurons during the early stages of neural differentiation [45], while DAPI was used to co-stain neuronal cell nuclei. The TTC-Exos were found to be distributed along the soma and axons of the neuronal cells (as depicted in Figure 4). Expectedly, the intensity and distribution of Cy5 (red) was significantly higher in the TTC-Exo samples (Figure 4B) than the Un-Exo (Figure 4A). A closer look at the distribution of TTC-Exos revealed that they were primarily located along the somal circumference of cells (Figure 4C). A semi-quantitative analysis was conducted to distinguish the distribution between both groups (Figure 4D). The TTC-Exo signal covered approximately twice as much area as the Un-Exo signal, indicating a notable improvement in cellular specificity and binding facilitated by the TTC protein. These results provide promising evidence that modifying Exos with the TTC fragment can significantly enhance the ability of the Exo drug delivery system to specifically target neurons in the PNS.

### 3.5. Modification of Exos with the TTC-Fragment Improves Transport into the PNS in a Mice Model

**Figure 5:**
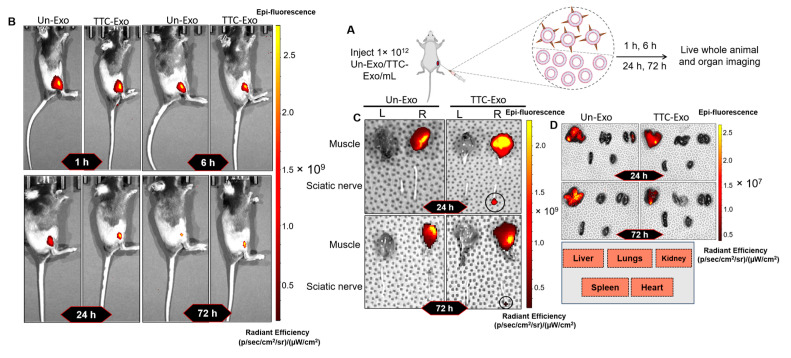
Bio-distribution and tissue imaging study for Un-Exos and TTC-Exos in vivo. (**A**) Schematic representation of intramuscular administration of labeled Exos, followed by IVIS imaging at different time points. (**B**) Whole-animal fluorescence images of Un-Exo and TTC-Exo groups at 1, 6, 24, and 72 h timepoints. (**C**) Epi-fluorescence images of the gastrocnemius muscle and sciatic nerve from the left and right regions of animals from Un-Exo and TTC-Exo groups that were dissected 24 and 72 h after administration (L = Left side/contralateral side and R = Right side/ipsilateral side). (**D**) Epi-fluorescence images of organs isolated from Un-Exo and TTC-Exo groups 24 and 72 h after administration.

Following an investigation into the improvement of target specificity to neuronal cell lines in vitro, an animal model was employed to evaluate the capacity for delivering Exos to the PNS. The experiment involved the injection of Un-Exos or TTC-Exos into the gastrocnemius muscle in the right hindlimb of mice, with the animals imaged at different time points (Figure 5A). The signal intensity in the injected tissue area decreased over time, as seen in images taken with the IVIS Lumina system (Figure 5B).

At 24 and 72 h, the animals were euthanized, and the gastrocnemius muscles and sciatic nerves were dissected and imaged. The IVIS lumina images showed intense fluorescence in the muscles for both Un-Exo and TTC-Exo groups (Figure 5B). TTC-Exos successfully bound to the sciatic nerves due to the TTC-fragment conjugated to Exo, as demonstrated by the fluorescence emission at the proximal end of the sciatic nerve at 24 and 72 h (Figure 5C). This indicates that TTC-Exos were successfully transported and bound to the sciatic nerves due to the TTC-fragments conjugated to Exos. After the animals were sacrificed, the tissue bio-distribution results in excised vital organs showed a low fluorescence intensity (10^7^ radiant efficiency) in the liver compared to the higher (10^9^ radiant efficiency) emission from muscle and nerve tissues for both groups, as demonstrated in Figure 5D. This low signal magnitude at the 72 h time point suggests that most of the injected Exos were primarily localized within muscles or transported along the peripheral nerves, rather than entering systemic circulation.

In summary, the results indicate that the TTC-Exo group was successful in delivering Exos to the PNS in the animal model.

### 3.6. IF Images of Muscle and Nerve Tissues Show Localization of TTC-Exo in PNS

**Figure 6:**
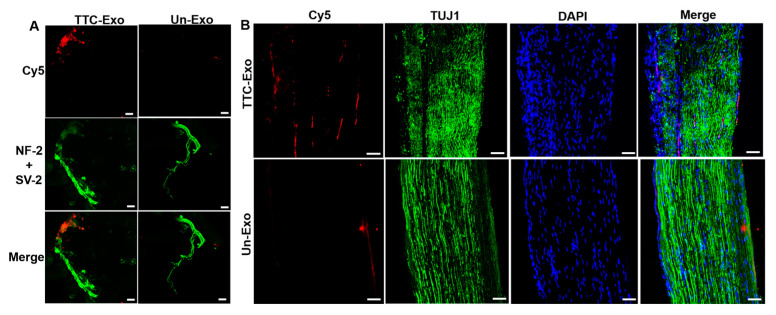
Immunostaining and fluorescence imaging study for Un-Exos and TTC-Exos in PNS tissues. (**A**) Immunostaining and CLSM images of the neuromuscular junction (NMJ) in the right gastrocnemius muscle fibers dissected from TTC-Exo and Un-Exo animal groups showing Cy5 (red) and neurofilament and synaptic vesicle-2 (green). (**B**) Immunostaining and CLSM images of sciatic nerves isolated from TTC-Exo and Un-Exo animal groups indicated by Cy5 (red), TUJ1 (green), and DAPI (blue). Scale bar = 50 µm.

Following the in vivo evaluation, the gastrocnemius muscle and sciatic nerve tissues from both TTC-Exo and Un-Exo groups were dissected and stained to track the progress of labeled Exo delivery after injection. The right-hindlimb gastrocnemius muscles were split into longitudinal fibers, and the neuromuscular junctions were visualized through antibody staining for neurofilament and the presynaptic vesicular marker (SV2), synaptophysin (Figure 6A). CLSM images clearly indicate significant accumulation of TTC-Exos in the motor neuron nerve bundles. In contrast, images from the Un-Exo group show a lack of affinity and binding of non-targeted Exos to the nerve bundles.

To assess if there was any indication of improved retrograde axonal transport of TTC-labeled Exos from muscles to peripheral nerves, the sciatic nerves were stained for TUJ1, while the nuclei were co-stained with DAPI, and imaged. The panel in Figure 6B shows a significant and even distribution of the labeled Exos throughout the nerve fibers of the TTC-Exo group. Conversely, the non-targeted Un-Exo group shows uneven distribution of Exos across the tissue and aggregation in a particular location along the nerve fiber. These observations suggest that TTC-Exos travel via a retrograde axonal transport mechanism along the PNS by binding to polysialoganglioside and presynaptic receptors on neurons, facilitating inter-neuronal transfer across peripheral nerves [46,47].

## 4. Discussion

The suboptimal recovery and regeneration of peripheral nerve tissues associated with peripheral neuropathies remain a clinical challenge. The complex structure of the PNS poses a challenge to effectively transport and deliver therapeutics. The nerve fascicles, consisting of axons and endoneurial blood vessels, are tightly packed by concentric layers of perineurial cells and the basement membrane matrix. These are then surrounded by the epineurium, which provides tensile strength [27,48]. This structure effectively blocks the transport of therapeutics in circulation from reaching the peripheral nerves. However, a potential solution to this problem is to deliver therapeutics to the peripheral nerves using retrograde axonal transport. This can be achieved by utilizing certain natural bacterial toxins that bind to neuronal receptors with high specificity and affinity. One such approach involves utilizing the tetanus toxin fragment C as a targeting protein to engineer a delivery system. Previous research has employed the tetanus toxin to alter PLGA-PEG-biotin nanoparticles for targeting neuroblastoma cells [49]. Moreover, TTC-targeted polyethyleneimine and chitosan nanocomplexes have successfully been shown to deliver non-viral gene therapeutics to primary dorsal root ganglion (DRG) and nerve crush injury models, respectively [20,22]. Nevertheless, the next generation of delivery platforms must prioritize carrying diverse payloads, enhancing systemic circulation, facilitating surface modification and synthesis, ensuring biocompatibility, as well as augmenting functional recovery.

The mechanism of action of the tetanus toxin, once it enters circulation, has shown that it binds tightly to the presynaptic membrane of motor neurons by interacting with arrays of presynaptic receptors and polysialogangliosides, particularly with G1b gangliosides [50]. These molecules, along with arrays of presynaptic receptors (APRs), containing complex lipid-based structures, like sphingomyelin, cholesterol, and GPI-anchored proteins, play a key role in high-affinity interaction [51]. It is then rapidly internalized via clathrin-dependent endocytosis and retrogradely transported through the endosomes in axons that are involved in signaling the spinal cord [52]. This unique pharmacokinetic property is exploited in this study by utilizing the 51.6 kDa C-terminus of the heavy chain of the tetanus neurotoxin, called fragment C [53], as a homing moiety by functionalizing it to Exo nanocarriers. Exos have certain significant clinical advantages as superior therapeutic carriers compared to synthetic nanoparticles, not only due to their low immunogenicity and safety profile but also naturally loaded Exos can be produced via genetic modifications to bypass issues of cargo degradation and low loading efficiency [33,54]. Additionally, the mechanical stiffness and structural integrity of the Exos in stressful environments, along with their ability to cross biological barriers like the blood–brain-and blood–nerve barriers, have proven them to be a promising next-generation delivery platform.

The engineered Exo delivery system has also shown interesting results in targeting PNS for various neuropathies. For example, in the case of inflammatory neuropathies, endoneurial cells dramatically upregulate intercellular adhesion molecules (ICAM)-1 (~10 fold) in response to pro-inflammatory cytokines [55,56]. Functionalized PLGA nanoparticles have shown promise for site-specific delivery by leveraging upregulated ICAM-1 [57]. Functionalized Exos will offer a safer and easy-to-modify alternative as a non-immunogenic cargo vehicle. Exos can be engineered to deliver bioactive mRNA, immunomodulatory factors, and chemokines that help suppress pro-inflammatory genes and facilitate repair in diabetic peripheral neuropathy (DPN) [58]. Studies have demonstrated that augmenting mesenchymal stem cell (MSC) derived Exo miR-146a can improve neurovascular function efficacy in a DPN mice model [59]. Additionally, cannabidiol-loaded MSC-derived Exo therapy has shown promise in alleviating mechanical and thermal hypersensitivity in a paclitaxel-induced neuropathic mice model [60]. While many studies have explored the therapeutic potential of functionalized Exos of diverse origins, these delivery systems must be enhanced by targeting strategies specific to the clinical pathology of neuropathy.

In this study, the in vitro neuronal cell uptake results demonstrated that the incorporation of TTC improved the internalization of Exos. After intramuscular injection in the hindlimb gastrocnemius, the results indicate a substantial presence of the modified TTC-Exo in the NMJ and nerve tissues. This increased accumulation could be due to the neurotoxin fragment interacting with the basal membrane components in the NMJ, with selective uptake facilitated by glycoproteins, such as nidogen-2 [61]. Reports suggest that the neurotoxin is trafficked along with neurotrophins signaling receptor TrkB, from the NMJ towards the spinal cord [41]. Evidence of this inter-neuronal transfer in vivo can be further seen in the fluorescence signal between the nerve fibers in the sciatic nerve section.

While this study offers a promising start to a targeted Exo-based drug delivery platform and its ability to transport to PNS, there are some areas that require further exploration. This report and others have demonstrated that Exos can be isolated in a high yield by treating RBCs with calcium ionophore chemical agent [35,62]. However, there is currently no developed and standardized purification or isolation method, which is a major challenge when transitioning to industrial scales [63,64]. The method used in this report has yielded pure Exos after repeated ultracentrifugation steps, but this can be cumbersome when working with multiple samples or groups. While the chemical modification protocol for producing TTC-functionalized exosomes is efficient, performing an in-depth characterization of the number of TTC molecules functionalized on the Exo surface has proven to be difficult, despite exploring multiple techniques. Although the NTA analysis shows an increase in Exo size and the overall negative charge has been recorded after TTC conjugation, the study is limited in understanding the exact change at a molecular level. Attempts to analyze the Exo surface functionalization with TTC using the Raman spectroscopic technique were also unsuccessful. Another challenge that needs to be addressed is the low drug loading efficiencies reported when loading Exos [65,66]. Conventional strategies such as lentiviral transfection and co-incubation methods have low loading capacities, while other exogenous methods with higher loading capabilities such as ultrasound and electroporation can damage vesicles or cause Exo aggregation [67,68]. These issues need to be addressed while exploring the therapeutic potential of this delivery platform using TTC-targeted Exos loaded with therapeutic bioactive drugs or substances in the PNS.

## 5. Conclusions

In conclusion, this study isolated RBC-derived Exos and employed a simple and effective click chemistry approach to successfully conjugate RBC-derived Exos with a TTC-fragment targeting protein and Cy5 dye for intercellular tracking. The results of the study showed that targeting neurotoxin enhanced the internalization of Exos within neuronal cells and improved the delivery of the Exos to the NMJ and nerve tissues, highlighting the development of a potential next-generation therapeutic delivery platform.

## Data Availability

The data that support the findings of this study are available from the corresponding author upon reasonable request.
